# Supplementary material for: Overexpression of MxbHLH18 Increased Iron and High Salinity Stress Tolerance in Arabidopsis thaliana
Source: Int J Mol Sci. 2022 Jul 20;23(14):8007. doi: 10.3390/ijms23148007 (PMC9319408; doi:10.3390/ijms23148007)
Supplement: Supplementary file 1 [file ijms-23-08007-s001.zip › Table S1.pdf]

Table S1. List of primers used in this study.

Primer Name	Primer sequence (5'→3')	Function
MxbHLH18-F	ATGGAGATTTCATCAAGAGGAG	Clone full length of *MxbHLH18*
MxbHLH18-R	TCACATGAACAGCTCAAAAGC	Clone full length of *MxbHLH18*
MxbHLH18-qF	GGGAGCTGTGCTCTTGATGT	q-PCR for *MxbHLH18*
MxbHLH18-qR	AGATCCTTCACCGAGAGGCT	q-PCR for *MxbHLH18*
Actin-F	ACACGGGGAGGTAGTGACAA	q-PCR for *Actin*
Actin-R	CCTCCAATGGATCCTCGTTA	q-PCR for *Actin*
AtIRT1-F	GCCCCGCAAATGATGTTACC	q-PCR for *AtIRT1*
AtIRT1-R	TCCAATGACCACCGAGTGAA	q-PCR for *AtIRT1*
AtFRO2-F	ATCGAAAGTCGCCACACCAT	q-PCR for *AtFRO2*
AtFRO2-R	GAGCCACAAACATCGCCAAG	q-PCR for *AtFRO2*
AtNAS2-F	CGACGTGGTTAATTCGGTGG	q-PCR for *AtNAS2*
AtNAS2-R	CATAACCACACACCGTCCGA	q-PCR for *AtNAS2*
AtACT2-F	TGTGCCAATCTACGAGGGTTT	q-PCR for *AtACT2*
AtACT2-R	TTTCCCGCTCTGCTGTTGT	q-PCR for *AtACT2*
AtZIF1-F	CGATATGCTGGGGCACTGA	q-PCR for *AtZIF1*
AtZIF1-R	CCGGTTATGGCAGACACACT	q-PCR for *AtZIF1*
AtOPT3-F	AAGCTTACTATAAACAGAGCCTTAGCT	q-PCR for *AtOPT3*
AtOPT3-R	ACAGGATCAACAAGGTACCTCCTC	q-PCR for *AtOPT3*
AtKIN1-F	TGTCAGAGACCAACAAGAATGC	q-PCR for *AtKIN1*
AtKIN1-R	CCGCATCCGATACACTCTTT	q-PCR for *AtKIN1*
AtCOR15a-F	GGCCACAAAGAAAGCTTCAG	q-PCR for *AtCOR15a*
AtCOR15a-R	CTTGTTTGCGGCTTCTTTTC	q-PCR for *AtCOR15a*
AtCBF1-F	GGCCGTAAGAAGTTTCGTGA	q-PCR for *AtCBF1*
AtCBF1-R	ATCGTCTCCTCCATGTCCAG	q-PCR for *AtCBF1*
AtCBF2-F	AACTCCGGTAAGTGGGTGTG	q-PCR for *AtCBF2*
AtCBF2-R	CGGCGTATAAATAGCCTCCA	q-PCR for *AtCBF2*
AtCBF3-F	ACAGAGGAGTTCGTCGGAGA	q-PCR for *AtCBF3*

AtCBF3-R	ACCAACGTCTCCTCCATGTC	q-PCR for *AtCBF3*
AtCOR15b-F	CACAACGTAGGAGCAAGCA	q-PCR for *AtCOR15b*
AtCOR15b-R	GAGGATGTTGCCGTCACTTT	q-PCR for *AtCOR15b*
