# Supplementary figures and images for: Overexpression of MxbHLH18 Increased Iron and High Salinity Stress Tolerance in Arabidopsis thaliana
Source: Int J Mol Sci. 2022 Jul 20;23(14):8007. doi: 10.3390/ijms23148007 (PMC9319408; doi:10.3390/ijms23148007)

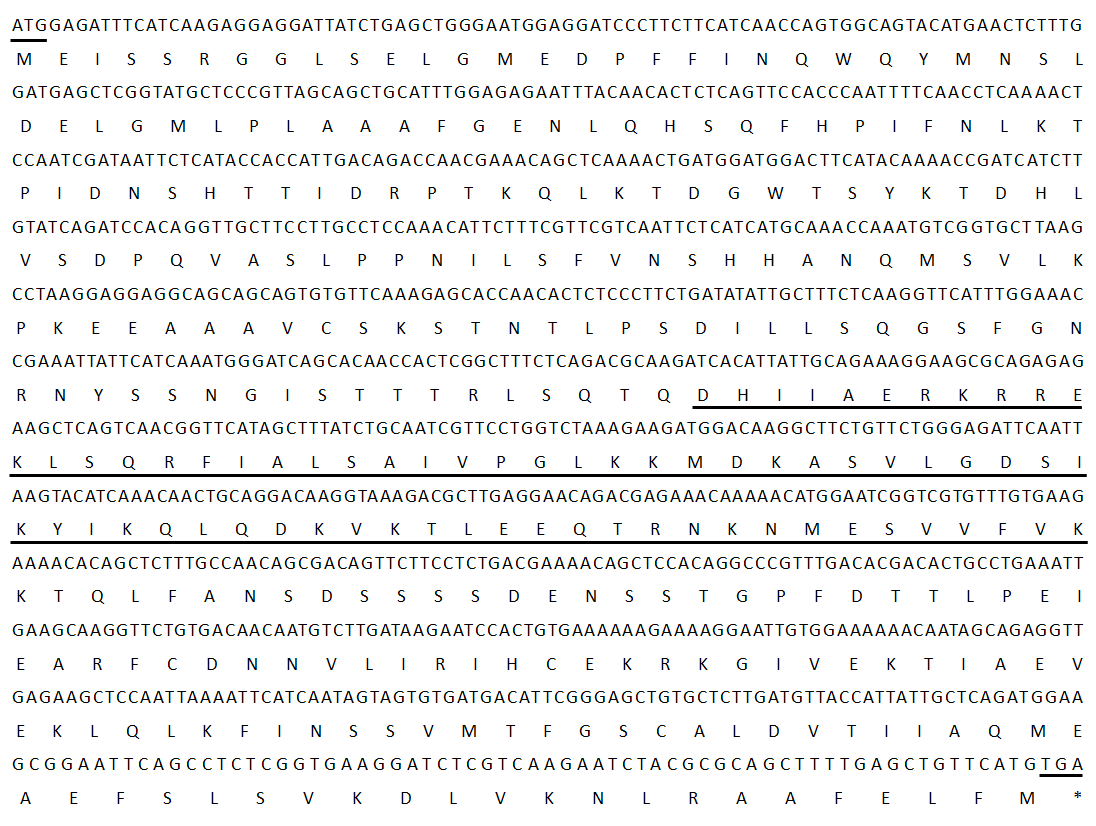

Supplement: Supplementary file 1 [file ijms-23-08007-s001.zip › Figure S1.tif]
